# Vulnerability and protective factors for PTSD in the academic community during the pandemic

**DOI:** 10.1186/s41155-025-00372-z

**Published:** 2026-01-15

**Authors:** Rachel Silva Machado Lana, Marta de Freitas Nudelman, Sarah Rocha Alves, Orlando Fernandes Junior, Raquel Menezes Gonçalves, Arthur Viana Machado, Rony Magalhães Martins, Liana Catarina Lima Portugal, Isabel de Paula Antunes David, William Berger, Fátima Cristina Smith Erthal, Eliane Volchan, Leticia de Oliveira, Mirtes Garcia Pereira

**Affiliations:** 1 Laboratory of Neurophysiology of Behavior, Department of Physiology and Pharmacology, Biomedical Institute, Federal Fluminense University, Rio de Janeiro, Brazil; 2 Laboratory of Neurobiology, Institute of Biophysics Carlos Chagas Filho, Rio de Janeiro, Brazil; 3 Laboratory of Cognitive Psychophysiology, Fluminense Federal University, Rio das Ostras, RJ Brazil; 4 Neurophysiology Laboratory, Department of Physiological Sciences, Roberto Alcantara Gomes Biology Institute, Biomedical Center, State University of Rio de Janeiro, Rio de Janeiro, Brazil; 5 Institute of Psychiatry of UFRJ, Department of Psychiatry and Mental Health, Federal University of Rio de Janeiro, Rio de Janeiro, Brazil

**Keywords:** PTSD, COVID-19, Academic community, Vulnerability factors, Protective factors, Mental health

## Abstract

**Background:**

The COVID-19 pandemic exposed individuals to potentially traumatic events, which can lead to the development of posttraumatic stress disorder (PTSD). However, only a portion of exposed people develop this disorder.

**Objective:**

This study aimed to examine the cross-sectional relationship between vulnerability factors and protective factors that can mitigate or exacerbate the development or severity of PTSD-related COVID-19 in the academic community.

**Methods:**

Members of the Brazilian academic community completed an online survey that included sociodemographic questions, the Traumatic Experiences During the COVID-19 Pandemic Questionnaire, the PTSD Checklist for the DSM-5, the Three-Item Loneliness Scale, the Fear of COVID-19 Scale, and the Life Orientation Test-Revised. Bivariate and multivariate logistic regression analyses were used to investigate the associations between vulnerability and protective factors and PTSD.

**Results:**

Logistic regression (*p* < 0.05) revealed that compared with professors/faculty members, undergraduates were 1.84 times more likely to develop PTSD. Additionally, each unit increase in the Fear of COVID-19 Scale score or the Three-Item Loneliness Scale score increased the likelihood of being in the group with a probable PTSD diagnosis by 25% and 18.8%, respectively, whereas optimism reduced the likelihood of a probable diagnosis of PTSD by 10.7%. Individuals who experienced two or three traumatic events were 2.1 times more likely to develop PTSD than those who experienced only one.

**Conclusion:**

This study highlights key vulnerability factors for PTSD in the academic community, including being an undergraduate student, experiencing multiple traumatic events, having a high level of fear of COVID-19, and experiencing loneliness. Conversely, optimism serves as a protective factor. These findings contribute to an enhanced understanding of PTSD, the identification of vulnerable groups, and the development of public policies, preventive strategies, and appropriate interventions for promoting mental health in the academic environment.

**Supplementary Information:**

The online version contains supplementary material available at 10.1186/s41155-025-00372-z.

## Introduction

The COVID-19 pandemic profoundly disrupted global public health (Burns & Horney, 2023; World Health Organization, 2024). This unprecedented situation increased general anxiety, depression, stress, and sleep disturbances across populations (Sher, 2020; Shigemura et al., 2020; Shreffler, 2020; Xiang et al., 2020). Infectious disease pandemics can be traumatic for individuals, and one possible outcome of exposure to potentially traumatic events is the development of posttraumatic stress disorder (PTSD) (Bridgland et al., 2021; Dimitrovska & Dimitrovska, 2024; Mondragon et al., 2023; Muysewinkel et al., 2024; Qiu et al., 2021).

PTSD develops following exposure to traumatic events involving actual or threatened death, serious injury, or sexual violence (American Psychiatric Association [APA], 2013). Such trauma is experienced or witnessed directly or indirectly when an individual learns that a loved one has suddenly died violently or accidentally, or it involves repeated or extreme exposure to aversive details of the traumatic event, usually due to professional duties. Clinical features of PTSD include intrusive thoughts, avoidance behaviors, negative changes in cognition and mood, and marked alterations in arousal and reactivity, with symptoms lasting more than one month and significantly impairing daily functioning (APA, 2013).

Early COVID-19 PTSD research primarily targeted frontline healthcare workers (Gama et al., 2022; Li et al., 2021a; Machado et al., 2023; Portugal et al., 2022), identifying prevalence rates as high as 21.7% in this population (Hill et al., 2022). Subsequent studies revealed substantial PTSD prevalence rates in broader populations, with rates of 30% among COVID-19 survivors (Bo et al., 2021; Janiri et al., 2021) and approximately 19% in the general population (Hoang et al., 2023). Women, in particular, were reported to be more vulnerable to pandemic-related PTSD symptoms (Liu et al., 2020).

The COVID-19 pandemic also significantly affected the academic setting, with studies showing an association between the pandemic and mental health problems among university faculty, staff, and students (Goldstein et al., 2023; Kirby et al., 2022; Sahu, 2020). These groups faced stressors such as social distancing, economic insecurity, and rapid adaptation to the online learning format (Sahu, 2020; Li et al., 2021a, b). One study reported that 36.3% of university faculty and staff members exhibited PTSD symptoms (Goldstein et al., 2023). University students appear even more vulnerable, with a meta-analysis documenting high levels of stress, anxiety, and depression (Li et al., 2021a, b) and another reporting a PTSD prevalence of 25% in this population (Hu et al., 2023).

PTSD has been described as the “second tsunami” in the context of the COVID-19 pandemic (Dutheil et al., 2021). However, researchers have expressed concerns that many PTSD studies conducted during the pandemic did not adequately assess trauma exposure (Husky et al., 2021; Muysewinkel et al., 2024). To address this, our study specifically anchored PTSD assessments to traumatic events that were directly associated with the COVID-19 pandemic, aligning with DSM-5 criteria A (American Psychiatric Association [APA], 2013). This methodological approach clarifies trauma-related distress and distinguishes it from general stress responses. This is an important contribution to the PTSD literature related to pandemics, given that PTSD symptom assessments should be anchored to a traumatic event in accordance with the DSM-5 criteria A for the diagnosis of PTSD. A significant contribution of this study is the investigation of psychological factors that may contribute to or protect individuals from developing PTSD related to COVID-19 trauma. Importantly, although exposure to a traumatic event is necessary for the development of PTSD, only some individuals exposed to such events develop the disorder. For example, an epidemiological study conducted before the COVID-19 pandemic in a Brazilian urban population (Luz et al., 2016) revealed that while 87% of the sample had been exposed to traumatic events, the conditional risk for developing PTSD was only 11.1%.

This study examined loneliness as a potential vulnerability factor and optimism as a protective factor. Loneliness, measured by the Three-Item Loneliness Scale (TILS; Hughes et al., 2004), is defined as a subjective discrepancy between desired and actual perceived social relationships (Cacioppo et al., 2015; Hawkley & Cacioppo, 2010). Prior research has identified loneliness as a vulnerability factor for psychological and physical health problems (Cacioppo et al., 2014). Longitudinal evidence has shown that higher PTSD symptoms and lower social support predict persistent loneliness trajectories among war veterans (Solomon et al., 2015). Similar findings in older adults indicate that changes in social and emotional loneliness track with changes in PTSD symptoms, suggesting a bidirectional relationship (Fox et al., 2021). Conversely, optimism, assessed by the Life Orientation Test-Revised (LOT-R; Scheier et al., 1994), reflects the general tendency to maintain positive future expectations and is widely recognized as beneficial for psychological resilience.

To better understand how these psychological factors influence PTSD vulnerability, it is helpful to consider concepts from the established stress-buffering model (Cohen & Wills, 1985). This model posits that social and psychological resources reduce the negative effects of stress by influencing how individuals appraise and cope with stressors. From this perspective, optimism may function as a cognitive buffer that shapes how individuals perceive potentially threatening situations and encourages more adaptive coping responses (Carver & Scheier, 2014). Conversely, loneliness can be viewed as a deficit in social resources that would otherwise protect against stress, thereby increasing susceptibility to trauma-related symptoms (Cohen & Wills, 1985).

In summary, this cross-sectional study examined the vulnerability and protective factors influencing the development of COVID-19-related PTSD in the academic community. We determined the prevalence of probable PTSD across university groups (academic staff, administrative staff, and graduate and undergraduate students) and investigated the influence of psychological factors, such as optimism and loneliness, as well as pandemic-related factors, such as fear of COVID-19 and the type and number of traumas.

## Materials and methods

### Study design and recruitment procedure

This study was part of the PSIcovidA project, a longitudinal study that was conducted to investigate the impact of the COVID-19 pandemic on the mental health of the Brazilian academic community. The present paper presents cross-sectional data collected over four months between March and June 2022.

Data were collected using a convenience snowball sampling technique. Although this approach may have introduced sampling bias—since participation could be influenced by internet access, social network connections, and willingness to complete an online survey—it was the most feasible option given the restrictions imposed by the pandemic. All the questionnaires were administered online and were sent by email and online messages and posted on social media. We also created an Instagram account (@projetopsicovida) and website (www.psicovida.org), both of which included a link to the online survey and information to increase understanding of the project.

## Psychometric instruments

### Sociodemographic and health questionnaire

The participants answered questions regarding their gender, age, race/ethnicity, occupation type and history of mental disorders. The terminology regarding race and ethnicity was in accordance with the official Brazilian census and the Brazilian Institute of Geography and Statistics (IBGE). The categories for race and ethnicity are defined on the basis of a spectrum of skin colors, ranging from very fair to very dark skin. The questionnaires utilize the following established IBGE categories: white, black, brown, yellow/Asian, and indigenous. Throughout the report, the black category encompasses both the IBGE black and brown categories.

## Questionnaire on traumatic experiences during the COVID-19 pandemic

This questionnaire consists of three items that investigate potentially traumatic events related to the COVID-19 pandemic. The items are as follows: (1) experiencing the imminent risk of death of a family member or close friend due to COVID-19; (2) being exposed to someone infected with COVID-19; and (3) being infected with COVID-19. All the items are in accordance with DSM-5 PTSD diagnostic criterion A, which involves direct or indirect exposure to death, threatened death, actual or threatened serious injury, or actual or threatened sexual violence. After completing the questionnaire, the participants had to choose their worst experience among the items listed above (index traumatic event) and how long ago the event occurred (less or more than one month). Only traumatic events lasting more than 30 days were considered in accordance with the DSM-5 criteria for PTSD.

The content validity of our survey of traumatic experiences during the COVID-19 pandemic was qualitatively examined based on the subjective judgments of PTSD experts (two psychiatrists and one psychologist) in accordance with the DSM-5 PTSD diagnostic criteria.

## Posttraumatic stress disorder checklist for the DSM-5 (PCL-5)

Posttraumatic stress symptoms were assessed via the Brazilian version (Lima et al., 2016) of the posttraumatic stress disorder checklist for the DSM-5 (PCL-5), an instrument originally developed by the National Center for PTSD (Weathers et al., 2013). This instrument measures the following four PTSD symptom clusters according to the DSM-5: intrusion, avoidance, negative alterations in cognition and mood, and alterations in arousal and reactivity. The checklist consists of 20 items, each rated on a 5-point Likert scale from 0 (not at all) to 4 (extremely). The mere presence of PTSD symptoms is not sufficient for a diagnosis; individuals must have been exposed to a traumatic event in accordance with DSM-5 criteria A for the development of PTSD. Therefore, the participants’ responses were associated with their worst COVID-19-related traumatic event. In addition, the participants were divided into two groups (those with or without a probable PTSD diagnosis) on the basis of their PCL-5 scores.

The ≥ 36 cutoff score employed in this study was derived from the Brazilian validation of the PCL-5, using the Structured Clinical Interview for DSM-5 (SCID-5-CV) as the reference standard (First & Williams, 2015), which demonstrated that this threshold provided optimal sensitivity and specificity for identifying PTSD in this population (Pereira-Lima et al., 2019). In their systematic review, Forkus et al. (2023) reported that cutoff scores maximizing diagnostic utility most often fall between 31 and 33 and highlighted that such variation is influenced by methodological and contextual factors, including sample characteristics and demographics, and that no universal cutoff score applies across all populations and settings. Additionally, international guidelines and validation studies conducted in different populations have recommended cutoffs within a broader range (31–38) for screening or provisional diagnosis, underscoring that the optimal threshold depends on the target population and the intended purpose of the assessment (Ashbaugh et al., 2016; Blevins et al., 2015; Bovin et al., 2016; Krüger-Gottschalk et al., 2017). Thus, the ≥ 36 cutoff adopted here reflects the most robust evidence available for the Brazilian population, while acknowledging that internationally, optimal thresholds vary according to context and purpose.

The psychometric properties of this scale include strong convergent and discriminant validity, very good-to-high test-retest reliability, and satisfactory-to-high internal consistency (Ashbaugh et al., 2016; Blevins et al., 2015).

The internal consistency in the present study, as assessed by Cronbach’s alpha, was high for the whole scale (0.952), as well as for the theoretical dimensions of the DSM-5 (criterion B = 0.909, criterion C = 0.797, criterion D = 0.904 and criterion E = 0.862).

### Fear of COVID-19 scale (FCV-19 S)

The Fear of COVID-19 Scale (FCV-19 S) is a seven-item self-rated scale developed and validated by Ahorsu et al. (2022) to assess the level of fear of COVID-19. This scale has been widely used in research to measure individuals’ fear of COVID-19 (Blázquez-Rincón et al., 2022). The questionnaire was translated into Brazilian Portuguese and validated by Cavalheiro and Sticca (2022) to ensure its applicability in the Brazilian context. In the questionnaire, participants are asked to rate each item on a five-point Likert-type scale ranging from 1 (“strongly disagree”) to 5 (“strongly agree”). The items are designed to capture dimensions of fear related to COVID-19, such as worry about personal health and physiological reactions, including heart racing and clammy hands. The seven items’ scores are added together to create an overall measure of fear via the standard procedure outlined in the literature (Ahorsu et al., 2022; Cavalheiro & Sticca, 2022). The total score ranges from 5 to 35 points, with higher scores indicating greater fear of COVID-19.

The internal consistency in the present study, as assessed by Cronbach’s alpha, was 0.871 for the whole scale.

## Three-Item loneliness scale

The Three-Item Loneliness Scale (TILS), developed and validated by Hughes et al. (2004), was designed as a concise and effective tool for assessing feelings of loneliness. Stemming from the UCLA Loneliness Scale (Russell et al., 1980), the TILS was adapted with the aim of reducing the number of items while retaining the evaluative capacity in measuring an individual’s perception of loneliness.

The selection of a brief instrument, such as the TILS, is a strategic methodological choice that addresses the need to minimize participant burden in large-scale studies, which is a common feature of pandemic-era research (Das et al., 2021). The TILS was specifically developed for efficient use in large surveys, and its three items were empirically selected, as they represent the psychometric core of the longer, 20-item Revised UCLA Loneliness Scale (R-UCLA) (Hughes et al., 2004). The suitability of the TILS for the Brazilian context is supported by multiple robust validations that confirm the reliability and validity of the original scale within the Brazilian context (Barroso et al., 2021; Mata et al., 2022). Indeed, the TILS itself has been successfully employed in the Brazilian context, including in a large-scale cross-national survey of university students during the COVID-19 pandemic (Prado et al., 2024).

Each item consists of a statement about perceived feelings of loneliness and is preceded by “How often do you feel…” with the following response options: (1) hardly ever, (2) some of the time and (3) often. For this unidimensional scale, higher scores correspond to higher levels of loneliness.

The internal consistency in the present study, as assessed by Cronbach’s alpha, was 0.775.

## Life orientation test

The Life Orientation Test-Revised (LOT-R) is a questionnaire designed to assess the way individuals perceive their Lives, in either a more optimistic or less optimistic manner. This test was initially developed by Scheier and Carver in 1985 and later revised by Scheier et al. in 1994. The scale was translated and validated in Portuguese by Bandeira et al. in 2002.

The use of the LOT-R is justified by its structure as a brief, efficient measure that is ideal for research protocols assessing multiple constructs and reduces survey fatigue (Scheier et al., 1994). A study conducted by Bastianello et al. (2014) confirmed the excellent psychometric properties of the instrument within a Brazilian sample and revealed its clear unidimensional structure and strong internal consistency (α = 0.80). The LOT-R has been applied in previous studies conducted during the COVID-19 pandemic in Brazil (Sousa et al., 2021; Almansa et al., 2024).

The LOT-R is a 10-item questionnaire consisting of a series of statements to which individuals respond on the basis of their agreement or disagreement. Each item is rated on a 5-point Likert scale from 0 (strongly disagree) to 4 (strongly agree). The test measures optimism, which reflects an individual’s tendency to expect positive outcomes and maintain a positive perspective in various life situations. The Brazilian scale version has been validated to have a unidimensional structure, and higher scores correspond to higher levels of optimism (Bandeira et al., 2002). The cultural adaptation and psychometric properties of the scale ensure its reliability and validity in assessing life orientation among Brazilian Portuguese individuals.

The internal consistency in the present study, as assessed by Cronbach’s alpha, was 0.820 for the whole scale.

### Participants

**Fig. 1:**
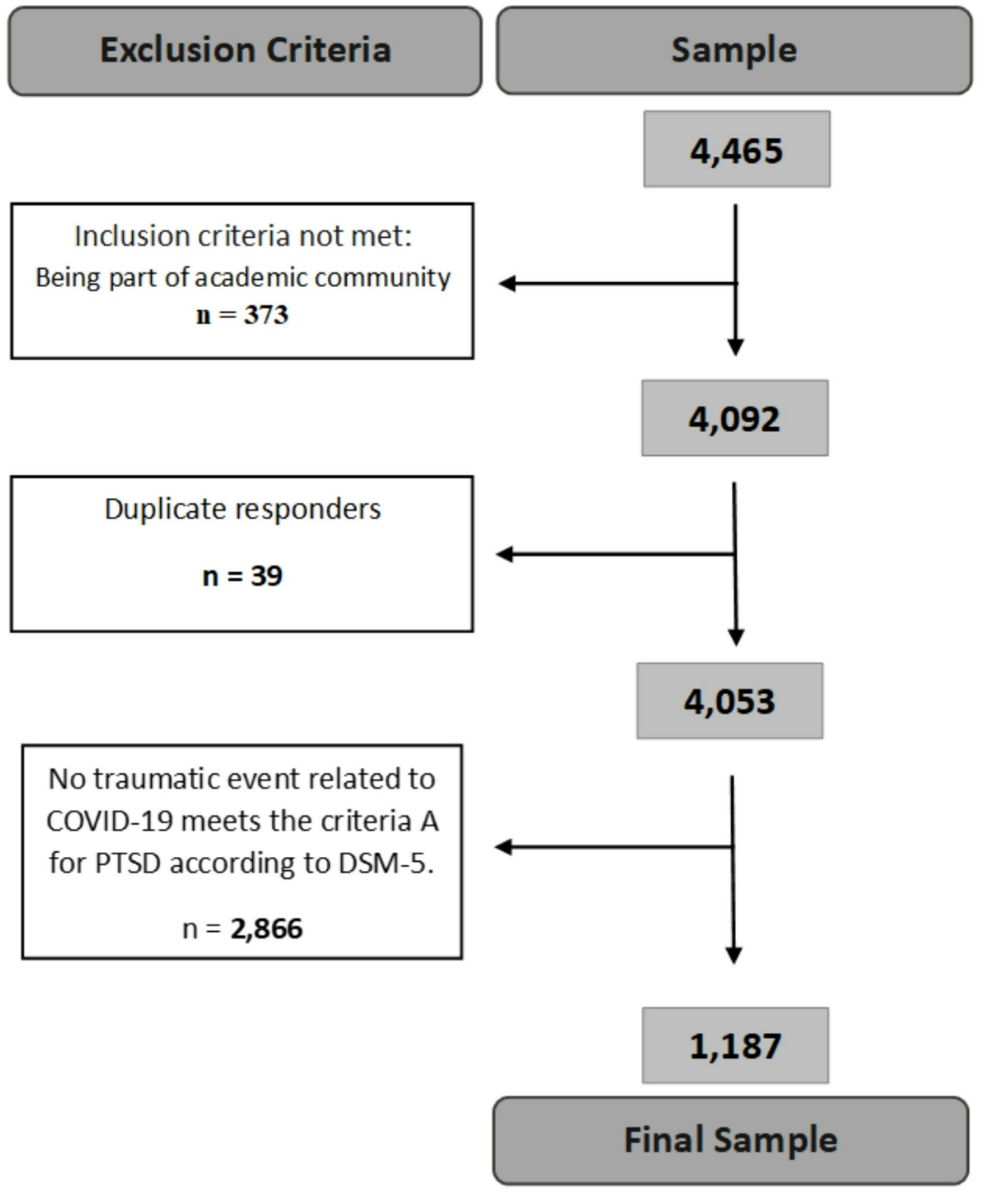
Flow diagram of the steps taken to obtain the final sample

In total, 4,465 respondents accessed the web survey. The inclusion criterion was being an academic community member from a university or research institute, e.g., a professor/faculty member, administrative staff member, graduate student, or undergraduate student, and its application resulted in a sample of 4053 participants. The exclusion criteria included individuals who were not academic community members from a university or research institute (*n* = 373), had duplicate responses (*n* = 39) and had not experienced a traumatic event related to COVID-19 in accordance with DSM-5 criteria A for PTSD (*n* = 2,866). The final sample consisted of 1,187 respondents from all 26 different states in Brazil. A flowchart presenting the steps followed to obtain the final sample is shown in Fig. 1. The characteristics of this sample are described in Table 1..

**Table 1.** Sample characteristics

	N (%)
	1.187 (100%)
Age - years	
18–29 years	355 (30%)
30–39 years	349 (29%)
40–49 years	260 (22%)
50–59 years	150 (13%)
60 years or older	73 (6%)
Gender	
Female	834 (70.3%)
Male	340 (28.6%)
Nonbinary	10 (0.8%)
Gender not declared	3 (0.3%)
Ethnic group	
Black	386 (32.5%)
White	759 (63.9%)
Yellow	11 (0.9%)
Indigenous	4 (0.3%)
Not declaring ethnic group	27 (2.3%)
Academic community	
Professor/Faculty members	394 (33.2%)
Administrative Staff	225 (19%)
Graduate Students	310 (26.1%)
Undergraduate Students	258 (21.7%)
Worst traumatic COVID-19 experience (index traumatic event)	
Experiencing the imminent risk of death of a family member or close friend due to COVID-19	518 (43.6%)
Being exposed to someone infected with COVID-19	300 (25.3%)
Being infected with COVID-19	369 (31.1%)
Previous diagnosis of a mental disorder	
No	736 (62%)
Yes	451 (38%)
Number of COVID-19-related traumatic events	
One traumatic event	316 (26.6%)
Two or three traumatic events	871 (73.4%)
Posttraumatic Stress Disorder (probable diagnosis)	
No	934 (78.7%)
Yes	253 (21.3%)
Posttraumatic Stress Disorder by academic segment (probable diagnosis)	
Professor/Faculty members	53 (13.45%)
Administrative Staff	49 (21,78%)
Graduate Students	73 (23,55%)
Undergraduate Students	78 (30,23%)
PCL score by academic segment	Mean (SD)
Professors/Faculty members	18.10 (16.03)
Administrative Staff	22.47 (16.50)
Graduate Students	21.81 (17.47)
Undergraduate Students	26.17 (19.21)
	Mean (SD)
Posttraumatic Stress Score	21.61 (17.38)
LOT-R score	14.66 (5.44)
Loneliness scale score	6.23 (2.25)
Fear of COVID-19 scale score	19.55 (5.66)

### Procedures

Individuals interested in participating in the study accessed the web survey by clicking on a provided link, which directed them to the Google Forms platform where the research protocol was hosted. Initially, a brief introduction outlined the study’s overall objectives and inquired whether the respondents belonged to the academic community. If the response was affirmative, the participants were directed to review the informed consent terms. Those who indicated that they were not part of the academic community were redirected to a page with instructions about mental health improvement.

After providing consent, the participants were asked to complete a sociodemographic questionnaire followed by questions addressing their traumatic experiences during the COVID-19 pandemic. The participants were subsequently asked to identify the event they considered the most traumatic (referred to as the trauma index) and complete the PCL-5 on the basis of that particular event. The participants subsequently completed the FCV-19 S, TILS, and LOT-R.

The participants spent approximately 20 min completing all the questionnaires, and after submitting their responses, they were provided with a text that offered supportive information on managing the psychological impact of the COVID-19 pandemic and a list of professional contacts for psychological support.

### Ethical approval and consent to participate

This study was approved by the Ethics Committee of the Fluminense Federal University (UFF) and the Brazilian National Research Ethics Commission (CONEP) under the Certificate of Presentation for Ethical Consideration (CAAE) number 52739721.0.0000.5243. All procedures were conducted in accordance with the Declaration of Helsinki. All the participants voluntarily agreed to participate in the study, and informed consent was obtained from each participant prior to data collection.

### Statistical analyses

Descriptive statistics are used to provide an overview of the sample characteristics. Participant age, gender, ethnic group, academic community category, worst traumatic COVID-19 experience, previous mental disorders, number of COVID-19-related traumatic events and probable PTSD diagnosis are described as percentages of total participants. Means and standard deviations are reported for the PCL-5, FCV-19 S, TILS and LOT-R scores. This information is shown in Table 1..

Bivariate and multivariable logistic regression analyses were conducted to investigate which variables were associated with the outcome variable (presenting or not presenting a probable PTSD diagnosis). The participants were grouped on the basis of the cutoff score of 36 points on the PCL-5 scale. One of the independent variables was the academic community category, which was subdivided into four groups (one factor with four levels): professors/faculty members, administrative staff, graduate students, and undergraduate students. Psychological factors were assessed via two independent continuous variables: (1) loneliness, measured by the TILS score, and (2) optimism, measured by the LOT-R score. Pandemic-related factors included three independent variables. Two variables were categorical, (1) the most traumatic COVID-19 experience and (2) the number of traumatic events experienced. The third variable, (3) fear of COVID-19, as assessed by the FCV-19 S score, was continuous.

For the analysis of psychological factors and pandemic-related factors, we conducted bivariate logistic regression analyses to examine the influence of each independent variable on the outcome. Our final multivariable logistic regression models were adjusted for a set of a priori selected potential confounding variables. The selection of these covariates was based on established theoretical models and extensive evidence identifying them as significant predictors of PTSD (Brewin et al., 2000). Specifically, gender was included as a covariate in all adjusted models because it is among the most robust and significant predictors of PTSD (Olff, 2017; Tolin & Foa, 2006). Therefore, controlling for gender is a methodological necessity to prevent its powerful and multifaceted influence from confounding the associations between our psychological and pandemic-related predictors and PTSD outcomes. Similarly, we adjusted for a history of previous mental disorders, which is recognized as one of the most potent pretraumatic vulnerability factors for PTSD development (Hapke et al., 2006). Age and ethnic group were also included as covariates to statistically account for developmental, life-stage, and sociocultural factors that are known to modulate trauma exposure, resilience, and symptom expression (Charak et al., 2014; Liu et al., 2021; Norris et al., 2001). For the academic community analyses, we report only the bivariate and final adjusted models, as there was only one independent variable of interest.

Complementary regression models were estimated separately for professors/faculty, administrative staff, graduate students, and undergraduate students to assess whether the effects of key predictors (loneliness, optimism, fear of COVID-19, and number of COVID-19–related traumatic events) varied across groups. The full results are provided in the Supplementary Material.

The statistical analysis included women, men, white individuals, and black individuals, with the exclusion of other groups because of the small number of respondents (nonbinary, gender not declared, yellow, indigenous, and ethnic group not declared). Therefore, the sample size for the statistical analyses was 1134.

Statistical analyses were conducted via SPSS 25 and Stata 12.0, with statistical significance set at *p* < 0.05.

## Results

### Sample characteristics

Among the 1187 participants enrolled, 834 (70.3%) were female, 340 (28.6%) were male, 10 (0.8%) were nonbinary, and 3 (0.3%) did not declare their gender. A total of 759 (63.9%) participants identified as white, 386 (32.5%) identified as black, 11 (0.9%) identified as yellow, 4 (0.3%) identified as indigenous, and 27 (2.3%) did not declare their ethnic group. Among the entire sample, 394 (33.2%) participants were faculty members or professors, 225 (19%) were administrative staff, 310 (26.1%) were graduate students, and 258 (21.7%) were undergraduate students (Table 1.).

The mean PCL-5 score for the entire sample was 21.61 points. Using a cutoff score of 36 points for the PCL-5 to identify individuals with a probable PTSD diagnosis, we observed that 21.3% (*n* = 253) of the participants had scores above the cutoff score (Fig. 2). A total of 518 participants (43.6%) considered the imminent risk of death of a family member or close friend due to COVID-19 as their worst traumatic experience during the pandemic (index traumatic event). Additionally, 300 participants (25.3%) reported exposure to someone infected with COVID-19 and 369 participants (31.1%) reported their own infection with COVID-19 as their worst traumatic experience related to the pandemic. Furthermore, 451 participants (38%) reported having a history of mental disorders.

In terms of the number of COVID-19-related traumatic events, 871 participants (73.4%) experienced more than one traumatic event. The mean LOT-R score of the sample was 14.66 points, the mean TILS score was 6.23 points, and the mean fear of COVID-19 score was 19.55 points.

### Academic community category as a predictor of PTSD

#### Prevalence of probable PTSD by academic group

The prevalence of probable PTSD varied across the different academic community categories. Undergraduate students showed the highest prevalence (30.23%), followed by graduate students (23.55%) and administrative staff (21.78%). Professors and faculty members had the lowest prevalence of probable PTSD, at 13.45% (Fig. 2).

A- Probable PTSD diagnosis B) Probable PTSD diagnosis according to the academic community.

among the total sample.

**Figure:**
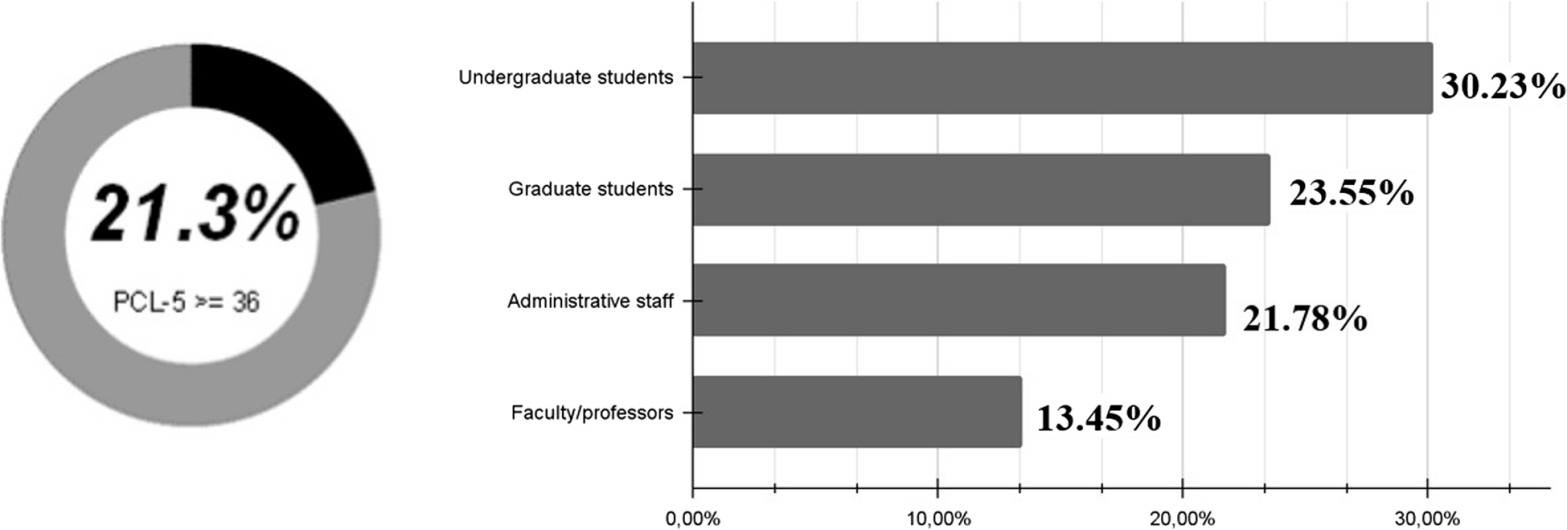
**a** The percentage of participants with a PCL-5 score > = 36 (cutoff score for a possible PTSD diagnosis) was 21.3% (*n* = 253) of the total sample. **b** Considering the percentage of individuals within each group, 13.45% (*n* = 53) of professors/faculty members, 21.78% (*n* = 49) of administrative staff, 23.55% (*n* = 73) of graduate students, and 30.23% (*n* = 78) of undergraduate students had scores above the cutoff score (≥ 36 points)

### Regression analyses for academic community category

To examine the likelihood of probable PTSD among the different groups, we conducted logistic regression analyses using professors/faculty members as the reference group (Table 2). In the bivariate (raw) model, administrative staff (OR = 1.77, *p* = 0.010), graduate students (OR = 1.91, *p* = 0.002), and undergraduate students (OR = 2.92, *p* < 0.001) all showed presented significantly higher odds of a probable PTSD diagnosis than professors/faculty members did.

After adjusting for potential confounders (age, gender, ethnic group, and history of previous mental disorders), the association remained statistically significant only for undergraduate students, who were 1.84 times more likely to have a probable PTSD diagnosis than professors/faculty members were (OR = 1.84, *p* = 0.030). The associations for administrative staff and graduate students were no longer statistically significant in the adjusted model.

**Table 2:** Bivariate and multivariable logistic regression showing the likelihood of probable PTSD among different academic community groups compared with professor/faculty members

	Bivariate Model (raw)			Multivariable Model (adjusted)		
	OR	95% CI	*p* value	OR	95% CI	*p* value
Professors/Faculty Members	Reference					
Administrative Staff	1.773	[1.145, 2.746]	0.010*	1.438	[0.895, 2.309]	0.133
Graduate Students	1.912	[1.281, 2.854]	0.002**	1.430	[0.890, 2.297]	0.139
Undergraduate Students	2.918	[1.954, 4.356]	***	1.837	[1.061, 3.178]	0.030*
Sociodemographic and Clinical Covariates						
Gender (Reference: Man) Woman				1.676	[1.160, 2.423]	0.006**
Race/Ethnicity (Reference: White) Black				1.524	[1.113, 2.086]	0.009**
Age (per year increase)				0.990	[0.973, 1.007]	0.244
Prior Mental Disorder (Reference: No) Yes				3.835	[2.824, 5.207]	***

*OR * Odds ratio, *CI * Confidence interval. Multivariable model adjusted for age, gender, ethnic group and previous mental disorders

*p < 0.05, **p < 0.01, ***p < 0.001

### Psychological factors as predictors of PTSD

#### Regression analyses for loneliness and optimism

We performed bivariate and multivariable logistic regression analyses to investigate which psychological variables were associated with the risk of a probable PTSD diagnosis (Table 3). In the final adjusted multivariable model, both loneliness and optimism remained significant predictors.

The analysis demonstrated that for each one-point increase in the loneliness score (TILS), the odds of having a probable PTSD diagnosis increased by 18.8% (OR = 1.188, *p* < 0.001). Conversely, for each one-unit increase in the optimism score (LOT-R), the odds of a probable PTSD diagnosis decreased by 10.7% (OR = 0.893, *p* < 0.001).

**Table 3:** Bivariate and multivariate logistic regression analyses showing psychological variables associated with increased and decreased likelihood of probable PTSD

	Bivariate Model			Multivariable Model (raw)			Multivariable Model (adjusted)		
	OR [95% CI] *p* value			OR [95% CI] *p* value			OR [95% CI] *p* value		
Loneliness (TILS)	1.267	[1.181,1.360]	***	1.208	[1.120,1.304]	***	1.188	[1.097,1.287]	***
Optimism (LOT-R)	0.868	[0.844,0.892]	***	0.877	[0.852,0.903]	***	0.893	[0.866,0.922]	***
Sociodemographic and Clinical Covariates									
Gender (Reference: Man) Woman							1.678	[1.145, 2.458]	0.008**
Race/Ethnicity (Reference: White) Black							1.686	[1.221, 2.328]	0.002**
Age (per year increase)							0.997	[0.0983, 1.011]	0.678
Prior Mental Disorder (Reference: No) Yes							2.974	[2.159, 4.097]	***

*OR * Odds ratio, *CI * Confidence interval. Multivariable model adjusted for age, gender, ethnic group, and previous mental disorders

*p < 0.05, **p < 0.01, ***p < 0.001

### Pandemic-related factors as predictors of PTSD

#### Regression analyses for pandemic-related factors

We next examined the association between pandemic-related factors and the likelihood of a probable PTSD diagnosis using logistic regression (Table 4). All associations were tested in both raw and adjusted models, controlling for age, gender, ethnic group, and history of mental disorders.

With respect to the index traumatic event, compared with those who reported “being infected with COVID-19” as their worst event, participants who reported “experiencing the imminent risk of death of a family member or close friend” had significantly greater odds of probable PTSD in the adjusted model (OR = 1.758, p = 0.007). The number of COVID-19-related traumatic events was also significantly associated with increased odds of a probable PTSD diagnosis. The final adjusted model revealed that individuals who experienced more than one traumatic event had a 2.1-fold greater risk of a probable PTSD diagnosis than did those who experienced only one traumatic event (OR = 2.099, p = 0.001). Finally, the fear of COVID-19 was also a significant predictor. The adjusted model showed an odds ratio of 1.250 (p < 0.001), indicating that for each one-unit increase in the scale score, the risk of a probable PTSD diagnosis increased by 25%. In the adjusted model, a prior mental disorder was associated with higher odds of PTSD, whereas older age was associated with lower odds (Table 4).

Across the three adjusted regression models, previous mental disorders consistently emerged as a significant predictor of PTSD. Being female and identifying as black were also significant predictors in the first two adjusted models. In the third adjusted model, age and previous mental disorders were significant, and although being female and being black were not statistically significant, their odds ratios indicated a higher risk for PTSD.

**Table 4:** Bivariate and multivariable logistic regression analyses of pandemic-related variables associated with the likelihood of a probable PTSD diagnosis

Pandemic-related Factors	Bivariate Model			Multivariable Model (raw)			Multivariable Model (adjusted)		
	OR	95% CI	*p* value	OR	95% CI	*p* value	OR	95% CI	*p* value
Index traumatic event									
Being infected with COVID- 19	Reference								
Being exposed to someone infected with COVID-19	1.128	[0.763,1.669]	0.546	1.712	[1.041,2.818]	0.034*	1.430	[0.844,2.421]	0.184
Experiencing the imminent risk of death of a family member or close friend due to COVID-19	1.370	[0.976,1.922]	0.068	1.711	[1.157,2.532]	0.007*	1.758	[1.165,2.653]	0.007*
Number of COVID-19-related traumatic events									
One traumatic event	Reference								
More than one traumatic event	1.404	[1.002,1.968]	0.049*	2.091	[1.361,3.213]	0.001*	2.099	[1.329,3.314]	0.001*
Fear of COVID-19	1.252	[1.210,1.295]	***	1.259	[1.216,1.303]	***	1.250	[1.205,1.297]	***
Sociodemographic and Clinical Covariates									
Gender (Reference: Man) Woman							1.266	[0.836, 1.916]	0.265
Race/Ethnicity (Reference: White) Black							1.341	[0.944, 1.906]	0.102
Age (per year increase)							0.966	[0.951, 0.981]	***
Prior Mental Disorder (Reference: No) Yes							3.186	[2.263, 4.485]	***

*OR * Odds ratio, *CI * Confidence interval. Multivariable model adjusted for age, gender, ethnic group and previous mental disorders

*p < 0.05, ***p < 0.001

## Discussion

The present study aimed to determine the percentage of participants with a probable PTSD diagnosis as a result of COVID-19-related trauma across different categories of the Brazilian academic community. This study revealed a point prevalence of probable PTSD of 21.3%. Undergraduate students had the highest point prevalence of probable PTSD (30.23%), followed by graduate students (23.55%), administrative staff (21.78%) and professors/faculty members (13.45%). The risk for a probable PTSD diagnosis was 1.84 times greater for undergraduates than for professors/faculty members. Furthermore, we investigated the cross-sectional relationship between risk factors and protective factors that can either mitigate or exacerbate the development of COVID-19-related PTSD in this sample. We observed that for each one-unit increase in the FCV-19 S and TILS scores, the probability of having a probable PTSD diagnosis increased by 25% and 18.8%, respectively, whereas for optimism, the probability decreased by 10.7%. Individuals who experienced two or three traumatic events were 2.1 times more likely to have a probable PTSD diagnosis than those who experienced only one COVID-19-related traumatic event were. The most prevalent index traumatic event was the “imminent risk of death of a family member or close friend due to COVID-19”.

The present study revealed a high prevalence of probable PTSD among undergraduate students, who were nearly twice as likely to meet the criteria as faculty members were, even after controlling for sociodemographic variables and a history of mental disorders. These findings reinforce evidence that undergraduates represent a particularly vulnerable group within the academic community, a vulnerability likely exacerbated by academic pressure and social disruption during the pandemic (Sheldon et al., 2021; Ahmed et al., 2023). In addition to these factors, social vulnerability, such as poor housing and poverty, has been shown to further increase the risk of mental disorders among students (Limone & Toto, 2022), and those who struggle with remote learning display higher prevalence rates of major depressive disorder and generalized anxiety disorder (Chirikov et al., 2020).

Research on PTSD related to the pandemic among university academic and administrative staff is scarce, with most studies concentrating on depression, anxiety, stress, and overall well-being (Al Miskry et al., 2021; Evanoff et al., 2020; Gloster et al., 2020; Jayman et al., 2022; Kotini-Shah et al., 2022). Our findings expand this literature by revealing high prevalence rates of probable PTSD among both academic and administrative staff. Previous studies have similarly documented a high prevalence of probable PTSD among university staff during the pandemic, reinforcing the magnitude of this issue (Goldstein et al., 2023; Mallhi et al., 2023). Pandemic-related stressors—such as the abrupt transition to remote work, the pressure to sustain productivity, and the challenges of balancing professional and personal demands—likely contributed to greater psychological strain in this population, resulting in individuals being more vulnerable to the development or increasing severity of PTSD. Importantly, unlike most prior studies that have assessed PTSD in relation to general life trauma, our study focused specifically on pandemic-related traumatic experiences and thus provides a more direct understanding of the psychological impact of COVID-19 in the academic community.

Loneliness is an important risk factor for PTSD, according to our findings. Loneliness is defined as the gap or contrast between the social relationships that an individual desires and those that they actually perceive they have (Panayiotou et al., 2023). This implies that individuals may experience loneliness even in the presence of others if their social interactions fail to satisfy their expectations or needs (Hawkley & Cacioppo, 2010). Humans depend on a safe and secure social environment to survive and prosper as a social species, and the experience of loneliness increases the desire to reestablish social connections while also increasing alertness to potential threats and intensifying feelings of vulnerability (Hawkley & Cacioppo, 2010). Adequate social connection is essential for mental health, which highlights that social disconnection can lead to multiple health risks (physical, mental, and cognitive). Indeed, subjective loneliness and the quality of one’s social network were found to be better predictors of mental health outcomes than the number of social contacts and living arrangements were (Beller & Wagner, 2018). The observed association between higher loneliness scores and a greater likelihood of PTSD may be understood in light of the social restrictions imposed during the COVID-19 pandemic to reduce the spread of the virus (World Health Organization, 2020), which limited interpersonal contact and likely intensified feelings of isolation and loneliness (Murayama et al., 2021). A potential biological mechanism linking loneliness to PTSD vulnerability is cortisol dysregulation, as increased prior-day feelings of loneliness have been associated with heightened cortisol awakening responses (Doane & Adam, 2010). In PTSD, acute cortisol elevations during trauma reminders can reinforce traumatic memory persistence (Elzinga et al., 2003), although importantly, cortisol alterations in PTSD are complex (Yehuda, 2006). Beyond biological factors, behavior helps explain the loneliness–PTSD link: loneliness—a perceived social deficit—often leads to reduced social engagement and difficulty mobilizing support (Choi et al., 2023). During the COVID-19 pandemic, disruptions in in-person contact and the receipt of emotional support were strongly tied to greater loneliness (Choi et al., 2023). Taken together, these findings outline a stress-amplifying pathway whereby social disconnection alters physiological reactivity and constrains help-seeking.

On the other hand, optimism is an important protective factor against PTSD. Our results suggested that for every one-point increase in the LOT-R score, the likelihood of a probable diagnosis of PTSD decreased by 10.7%. Our findings are in line with those of other studies showing that optimism plays an important role in promoting adaptive coping strategies in adverse and stressful situations (Agbaria & Abu Mokh, 2022; Santos et al., 2022). A meta-analysis confirmed that optimism protects against the development of PTSD in trauma-exposed individuals (Gallagher et al., 2020). Optimism is defined as the tendency to have positive expectations for the future (Scheier et al., 1994). This means that optimistic individuals expect to experience more positive outcomes than negative ones. This positive attitude may have beneficial effects on many facets of life, including mental health, physical health, and overall well-being (Bandeira et al., 2002; Nunes et al., 2023; Scheier et al., 1994). Optimism buffers stress through cognitive appraisal processes that construe stressors as manageable challenges and foster flexible, approach-oriented coping (Nes & Segerstrom, 2006). Optimists are more likely to interpret stressors as manageable challenges, which facilitates the use of flexible, approach-oriented coping strategies, such as problem-solving and seeking support (Nes & Segerstrom, 2006). Additionally, neuroendocrine evidence has shown that pessimists exhibit elevated diurnal cortisol on high-stress days, whereas optimists display faster cortisol recovery after acute stress (Jobin et al., 2014; Puig-Perez et al., 2015). Taken together, these converging mechanisms help explain why optimism may buffer against PTSD. In our sample, this protective role was likely expressed through more positive appraisals and flexible coping, which allowed participants to manage pandemic stressors more effectively.

Although gender, race and previous mental disorders were not variables of primary interest and were included as confounding covariates in our models, it is crucial to recognize that they represent well-established axes of psychosocial vulnerability that likely shaped individuals’ differential responses to the pandemic. The literature consistently demonstrates that women in Brazil faced aggravated psychosocial vulnerabilities during the crisis, potentiated by the disproportionate burden of domestic work and caregiving (Rocha et al., 2025) and a documented surge in gender-based violence and femicides during social isolation periods (Tavares, 2021; World Bank, 2020). Similarly, controlling for race/ethnicity does not capture the full scope of structural racism, which rendered the pandemic a disproportionately lethal event for black populations in Brazil, who experienced higher excess mortality rates and greater socioeconomic disruption (Marinho et al., 2021). Furthermore, a history of mental disorders, a potent pretraumatic vulnerability factor, likely sensitized individuals to the chronic stressors of the pandemic, which may have acted not only as a new traumatic event but also as an amplifier of preexisting conditions (Murphy et al., 2021).

It is essential to interpret these findings within the Brazilian sociocultural context during the pandemic, which was marked by overlapping crises. Brazil experienced one of the largest sanitary and hospital collapses in its history, becoming the epicenter of the pandemic in Latin America (The Lancet, 2020), and this public health crisis was further aggravated by deep social inequalities (Fiocruz, 2021) and intense political instability. Polarization, the spread of misinformation, and conflicting messages from government leadership against social distancing measures undermined adherence to public health guidelines, creating a climate of insecurity and chronic stress (Camacho et al., 2024). Within the academic environment, the abrupt transition to emergency remote learning highlighted the country’s profound digital infrastructure inequalities, as students and professors were forced to adapt rapidly, often without adequate training or access to technological resources (Gusso et al., 2020). This convergence of factors created an exceptionally adverse environment that likely intensified psychological distress and helps explain the high rates of probable PTSD observed, particularly the heightened vulnerability among students.

A significant strength of our study was the assessment of PTSD symptoms anchored to COVID-19-related trauma, which is in accordance with DSM-5 criteria A for PTSD. It is essential to anchor the assessment of PTSD symptoms to be related to a DSM-defined traumatic event (Van Overmeire, 2020) to ensure accurate interpretations of PTSD symptoms and their distinction from general distress (Asmundson & Taylor, 2021). A recent review revealed that 70% of studies on posttraumatic stress during the COVID-19 pandemic did not provide any indication of an index event to which PTSD symptoms were attributed (Muysewinkel et al., 2024). Without this specification, it becomes challenging to determine whether the symptoms are genuinely related to a traumatic experience or are due to other stressors. Additionally, more than half of the studies (54.1%) used scales based on the DSM-IV criteria (Muysewinkel et al., 2024), which is an older version of the DSM. The DSM-5, which includes revised criteria for PTSD, is the most recently updated version. The use of outdated criteria can lead to inconsistencies in diagnosis and may not fully capture the current understanding of PTSD symptoms and their manifestations.

Our data revealed that the worst pandemic traumatic experience with the greatest prevalence was the imminent risk of death of a family member or close friend due to COVID-19 (43.6%). These results underscore the critical psychological toll of the pandemic, particularly the fear of losing loved ones, as highlighted by previous studies (Caci et al., 2024; Portugal et al., 2022). Additionally, compared with experiencing only one traumatic event, experiencing more than one traumatic event was associated with a 2.1-fold greater risk of a probable PTSD diagnosis. Indeed, exposure to different types of potentially traumatic events has been described as a factor that might increase the risk of developing PTSD (Al Jowf et al., 2022; Kolassa et al., 2010).

Finally, the positive association between fear of COVID-19 and the likelihood of a probable diagnosis of PTSD was expected because fear is a core component of PTSD given its involvement in the body’s response to a traumatic event, as it leads to increased arousal and anxiety levels (Vasterling & Verfaellie, 2009; Zoellner et al., 2014). Additionally, PTSD is a disorder of the brain’s fear circuitry that is characterized by the dysregulation of neurobiological, psychophysiological, and emotional processing (Shvil et al., 2013). Emotional reactions are associated with the foundational mechanisms of information processing, such as attention and memory, which are modified in individuals with PTSD (Zoellner et al., 2014). Therefore, excessive fear has a negative impact on mental health, leading to increased anxiety levels and the adoption of maladaptive coping strategies, such as avoidance and denial, which increase vulnerability to psychological distress in the context of fear of COVID-19 (Oti-Boadi et al., 2022). Our findings regarding loneliness and the fear of COVID-19 have important clinical and public health implications. The observed 18.8% increase in the odds of probable PTSD for each one-point increase on the loneliness scale (TILS), although seemingly modest, represents meaningful cumulative vulnerability, with a four-point difference nearly doubling the odds. This magnitude highlights loneliness as a clinically relevant risk factor, comparable to other established predictors, such as low perceived social support, which has been consistently identified in meta-analytic research as a robust predictor of PTSD following trauma (Ozer et al., 2003). Notably, the effect size for loneliness in our sample approached that of fear of COVID-19, highlighting that psychological distress can be as impactful as direct fear of the virus itself. From a clinical perspective, these results indicate that loneliness should be seen not merely as a byproduct of trauma but as an independent and modifiable risk factor and therefore a meaningful target for preventive and therapeutic interventions. Screening for loneliness in academic settings could help identify at-risk populations who might benefit from targeted psychosocial support. At the public health level, particularly within universities, these findings underscore the need for institutional initiatives that foster social connection and reduce isolation, which could serve as primary prevention strategies to mitigate trauma-related distress.

The evidence presented here suggests concrete directions for improving mental health support in universities. The heightened vulnerability of undergraduate students suggests that this group should be prioritized for proactive screening, especially during high-stress academic periods. To maximize impact, such efforts must be paired with accessible and diverse mental health resources, including online options, to reduce barriers to care. Taken together, these implications call for a shift toward proactive, institution-wide approaches in which mental health promotion is recognized as a shared responsibility integral to the academic mission.

Notably, the additional parameters not examined in this study may also contribute to framing the pandemic as a significant source of trauma. The classification of COVID-19 as a disease-associated traumatic event extends beyond the direct experience of illness to encompass a range of secondary stressors. Government-imposed lockdowns, which involved prolonged social isolation and the disruption of daily routines, as well as the pervasive consumption of pandemic-related news media, have been identified as common stressors associated with PTSD-like symptoms during this period (Bridgland et al., 2021). Financial hardship and economic instability further exacerbated psychological distress for many individuals (Villatoro et al., 2022). Moreover, for those who became ill, hospitalization itself is recognized as a substantial risk factor for posttraumatic stress symptoms, particularly in patients who required intensive care (Janiri et al., 2021). Collectively, these contextual stressors created a sustained environment of threat that likely heightened vulnerability to PTSD across the population.

### Limitations

This research has several limitations. This prospective cross-sectional study is susceptible to memory bias, as participants may not be able to accurately remember experiences or events. Data were collected via convenience snowball sampling, which involved a survey link sent by email and WhatsApp. As a nonprobabilistic method, this sampling method limits representativeness and warrants caution when generalizing the findings. Although this method was practical during pandemic restrictions, the reliance on participants’ networks likely introduced selection bias and resulted in a less representative sample. Reliance on self-reports may introduce measurement error, as participants can under- or overreport behaviors and symptoms because of recall or social desirability biases; this is commonly observed in online surveys. Trauma exposure was also self-reported; although the questionnaire followed the DSM-5 criteria and included a trauma index for Criterion A events, structured clinical interviews remain the gold standard (International Society for Traumatic Stress Studies, n.d.) but were not feasible during pandemic restrictions. Additionally, all the predictors and outcomes were measured from the same source at a single time point, raising concerns of common-method variance. To reduce this risk, we implemented procedural remedies (Podsakoff et al., 2003): ensuring anonymity, separating questionnaires into distinct blocks, and minimizing evaluation apprehension. While these strategies mitigate bias, they cannot fully eliminate it and should be considered when the findings are interpreted. Finally, certain demographic categories (for example, nonbinary persons and indigenous participants) were not included in the analysis because of limited sample sizes. While this decision was methodologically necessary to maintain statistical reliability, it limits the representativeness of the findings and reduces inclusivity.

## Conclusion

This study contributes to the pandemic literature by examining PTSD symptoms in a Brazilian academic population using DSM-5–based assessments that directly link reported symptoms to a specific criterion A trauma. Such methodological rigor is important, as most COVID-19 studies do not specify an index of trauma, which risks the overestimation of PTSD by conflating it with general distress (Muysewinkel et al., 2024). Beyond estimating prevalence, this study highlights process-oriented predictors that help explain the psychological mechanisms underlying trauma responses. These findings provide guidance for university administrators and policy makers and underscore the need for proactive strategies that foster social connection and build resilience, such as programs to reduce loneliness and cultivate optimism. Given the high prevalence of PTSD across students, faculty, and administrative staff, initiatives should encompass the entire academic community. Future research should employ longitudinal designs, test interventions that strengthen optimism and reduce loneliness, and incorporate qualitative or mixed-method approaches to capture lived experiences of trauma and coping. These approaches can enrich quantitative evidence and guide more effective support for vulnerable groups in universities.

## Supplementary Information

Supplementary Material 1

## Data Availability

The datasets used and/or analyzed during the current study are available from the corresponding author upon reasonable request.

## Abbreviations

CAAE: Certificate of Ethical Appreciation Presentation
CONEP: National Research Ethics Commission
DSM-IV: Diagnostic and Statistical Manual of Mental Disorders, Fourth edition
DSM-5: Diagnostic and Statistical Manual of Mental Disorders, Fifth Edition
FCV-19S: Fear of COVID-19 Scale
IBGE: Brazilian Institute of Geography and Statistics
LOT-R: Life Orientation Test-Revised
PCL-5: Posttraumatic Stress Disorder Checklist for Diagnostic and Statistical Manual of Mental Disorders, Fifth Edition
PTSD: Posttraumatic stress disorder
SPSS: Statistical Package for the Social Sciences
TILS: Three-Item Loneliness Scale
UCLA: University of California Los Angeles
UFF: Federal Fluminense University
