# Supplementary material for: Vulnerability and protective factors for PTSD in the academic community during the pandemic
Source: Psicol Reflex Crit. 2026 Jan 15;39:1. doi: 10.1186/s41155-025-00372-z (PMC12804594; doi:10.1186/s41155-025-00372-z)
Supplement: Supplementary file 1 — Supplementary Material 1 [file 41155_2025_372_MOESM1_ESM.docx]

SUPPLEMENTAL MATERIAL

**Psychological factors**

To determine whether the effects of psychological predictors varied across segments of the academic community, we estimated multivariable logistic regression models separately for professors/faculty, administrative staff, graduate students, and undergraduate students (Table S1), adjusting for the same covariates as in the primary model. Overall, the pattern of effects was consistent with the main analysis (Table 2): higher loneliness scores were associated with higher odds of probable PTSD, whereas higher optimism scores were associated with lower odds.

Among professors/faculty, loneliness remained positively associated with probable PTSD (OR = 1.297, p = 0.001), indicating an approximate 30% increase in the odds per point increase on the TILS, whereas optimism was inversely associated (OR = 0.910, p = 0.007). A similar pattern was observed for graduate students, with loneliness showing a positive association (OR = 1.195, p = .016) and optimism a negative association (OR = 0.888, p < .001). Among administrative staff, optimism remained protective (OR = 0.906; p = 0.009), whereas the association with loneliness did not reach statistical significance (OR = 1.054; p = 0.584). For undergraduate students, optimism was again inversely associated with probable PTSD (OR = 0.872, p < 0.001), whereas the estimate for loneliness was directionally positive but failed to reach statistical significance (OR = 1.147, p = 0.172). Overall, the findings were consistent across groups, with optimism demonstrating a protective association with probable PTSD across all categories, and loneliness was positively associated with PTSD among professors and graduate students and showed directionally similar but nonsignificant effects among administrative staff and undergraduates — likely reflecting smaller sample sizes in these groups rather than substantive differences in effect. (Table S1.)

**Table S1. Multivariable logistic regression analyses of psychological variables associated with the likelihood of probable PTSD, stratified by academic segment.**

| **Predictor** | **Faculty/Professors** | **Administrative Staff** | **Graduate Students** | **Undergraduate Students** |
| --- | --- | --- | --- | --- |
|  | **OR [95% CI] p value** | **OR [95% CI] p value** | **OR [95% CI] p value** | **OR [95% CI] p value** |
| Loneliness (TILS) | 1.297 [1.110, 1.517] 0.001** | 1.054 [0.874, 1.271] 0.584 | 1.195 [1.033, 1.383] 0.016* | 1.147 [0.942, 1.396] 0.172 |
| Optimism  (LOT-R) | 0.910 [0.850, 0.975] 0.007** | 0.906 [0.842, 0.975] 0.009** | 0.888 [0.836, 0.942]*** | 0.872 [0.819, 0.927] *** |

*Note.* OR = odds ratio; CI = confidence interval. All models were adjusted for age, gender, ethnic group, and previous mental disorders. **p* < 0.05. ***p* < 0.01. ****p* < 0.001.

**Pandemic-related factors**

To examine whether pandemic-related predictors varied across segments of the academic community, we fit adjusted logistic regression models separately for professors/faculty, administrative staff, graduate students, and undergraduate students (Table S2). Stratified analyses by group were not feasible for the predictor type of index trauma because the sample size became too small for certain trauma categories, which would compromise the reliability of the estimates.

Fear of COVID-19 emerged as a robust correlate of probable PTSD across all groups, with each unit increase in the scale associated with higher odds among professors/faculty (OR = 1.282), administrative staff (OR = 1.236), graduate students (OR = 1.310), and undergraduate students (OR = 1.230; all p < .001). When the impact of cumulative trauma was assessed, experiencing more than one COVID-19-related traumatic event significantly predicted higher odds among administrative staff (OR = 3.088, p = .036). Although the odds were also elevated in the other groups, these effects did not reach statistical significance (graduate students: OR = 2.326, p = .089; professors/faculty: OR = 1.383, p = .472; undergraduate students: OR = 1.772, p = .229). Given that the effect was consistently in the same direction across groups, the absence of statistical significance likely reflects reduced statistical power due to sample stratification rather than substantive differences in effect (Table S2).

**Table S2. Multivariate logistic regression analyses of pandemic-related variables associated with the likelihood of a probable PTSD diagnosis, stratified by academic group.**

| **Predictor** | **Faculty/Professors** | **Administrative Staff** | **Graduate Students** | **Undergraduate Students** |
| --- | --- | --- | --- | --- |
|  | **OR [95% CI] p value** | **OR [95% CI] p value** | **OR [95% CI] p value** | **OR [95% CI] p value** |
| **Index Traumatic Event** |  |  |  |  |
| Being infected with COVID-19 | Reference | |  |  |
| Being exposed to someone infected with COVID-19 | 0.804 [0.263, 2.454] 0.701 | 2.810 [0.792, 9.972] 0.110 | 1.807 [0.628, 5.196] 0.272 | 0.992 [0.352, 2.796] 0.988 |
| Experiencing the imminent risk of death of a family member or close friend due to COVID-19 | 0.807 [0.369,1.764] 0.590 | 4.842 [1.775, 13.214] 0.002* | 2.704 [1.176, 6.221] 0.019* | 1.090 [0.464, 2.563] 0.843 |
| **Number of Traumatic Events** | | |  |  |
| One traumatic event | Reference |  |  |  |
| More than one traumatic event | 1.383 [0.572, 3.341] 0.472 | 3.088 [1.079, 8.835] 0.036* | 2.326 [0.881, 6.146] 0.089 | 1.772 [0.698, 4.501] 0.229 |
| **Fear of COVID-19** | 1.282 [1.189, 1.382] *** | 1.236 [1.140, 1.340] *** | 1.310 [1.206, 1.422] *** | 1.230 [1.139, 1.328] *** |

OR = odds ratio; CI = confidence interval. All models were adjusted for age, gender, ethnic group, and history of mental disorders. **p* < 0.05. ****p* < 0.001.
